# Interactive Effects of Microbial Fertilizer and Soil Salinity on the Hydraulic Properties of Salt-Affected Soil

**DOI:** 10.3390/plants13040473

**Published:** 2024-02-07

**Authors:** Xu Yang, Ke Zhang, Tingting Chang, Hiba Shaghaleh, Zhiming Qi, Jie Zhang, Huan Ye, Yousef Alhaj Hamoud

**Affiliations:** 1 College of Hydrology and Water Resources, Hohai University, Nanjing 210024, China; yangx@hhu.edu.cn (X.Y.); yousef-hamoud11@hhu.edu.cn (Y.A.H.); 2 The National Key Laboratory of Water Disaster Prevention, Hohai University, Nanjing 210024, China; 3 China Meteorological Administration Hydro-Meteorology Key Laboratory, Hohai University, Nanjing 210024, China; 4 Yangtze Institute for Conservation and Development, Hohai University, Nanjing 210024, China; 5 Key Laboratory of Water Big Data Technology of Ministry of Water Resources, Hohai University, Nanjing 210024, China; 6 College of Agricultural Science and Engineering, Hohai University, Nanjing 211100, China; changtt@hhu.edu.cn (T.C.); zhangjiejxd@hhu.edu.cn (J.Z.); 7 College of Environment, Hohai University, Nanjing 210024, China; hiba-shaghaleh@njfu.edu.cn; 8 Department of Bioresource Engineering, McGill University, Montreal, QC H9X 3V9, Canada; zhiming.qi@mcgill.ca; 9 Nanjing Hydraulic Research Institute, Nanjing 210029, China; ns201800@163.com

**Keywords:** coastal saline soil, effective microorganisms, secondary salinization soil, soil water availability, soil water holding capacity, soil water supply capacity

## Abstract

Significant research has been conducted on the effects of fertilizers or agents on the sustainable development of agriculture in salinization areas. By contrast, limited consideration has been given to the interactive effects of microbial fertilizer (MF) and salinity on hydraulic properties in secondary salinization soil (SS) and coastal saline soil (CS). An incubation experiment was conducted to investigate the effects of saline soil types, salinity levels (non-saline, low-salinity, and high-salinity soils), and MF amounts (32.89 g kg^−1^ and 0 g kg^−1^) on soil hydraulic properties. Applied MF improved soil water holding capacity in each saline soil compared with that in CK, and SS was higher than CS. Applied MF increased saturated moisture, field capacity, capillary fracture moisture, the wilting coefficient, and the hygroscopic coefficient by 0.02–18.91% in SS, while it was increased by 11.62–181.88% in CS. It increased soil water supply capacity in SS (except for high-salinity soil) and CS by 0.02–14.53% and 0.04–2.34%, respectively, compared with that in CK. Soil available, readily available, and unavailable water were positively correlated with MF, while soil gravity and readily available and unavailable water were positively correlated with salinity in SS. Therefore, a potential fertilization program with MF should be developed to increase hydraulic properties or mitigate the adverse effects of salinity on plants in similar SS or CS areas.

## 1. Introduction

Soil salinization has become a serious threat to global agricultural production and food security, leading to the alteration or disruption of agricultural land productivity [1,2]. Secondary salinization soil (SS) and coastal saline soil (CS) are important types of saline soil in China. To improve crop yield, organic and inorganic fertilizers are typically applied to increase agricultural productivity, while nitrate from fertilization has been used more than that required for crop cultivation [3,4]. Subsequently, considerable nitrate accumulation in the soil results in secondary soil salinization [5,6]. Meanwhile, rising sea levels, seawater intrusion, and upward saline groundwater movement in coastal areas cause coastal soil salinization [7]. The aforementioned soil salinization types are considered major factors that limit the sustainable development of the agricultural ecological environment in China and worldwide [8,9,10,11]. Therefore, saline soil remediation has become an important strategy for improving agricultural production to achieve global food security. To properly remediate and utilize saline soil, understanding the response of soil hydraulic properties to salinity levels is crucial because soil hydraulic properties are important factors that influence plant growth and salt migration [12,13,14].

Information on soil hydraulic properties (e.g., soil water holding capacity, saturated moisture, field capacity, permanent wilting point, soil water supply capacity, and soil water availability) is crucial for solving relevant issues in the hydrological, soil, agricultural, ecological, and environmental fields [15,16,17,18,19,20]. Moreover, soil hydraulic properties are the governing factors for the retention and transportation of salt and nutrients, particularly in soil salinization areas [21]. However, salts in soil water also affect soil hydraulic properties because they restrict plant roots from extracting water from the surrounding soil [22]. Hence, improving soil hydraulic properties has become an urgent problem in soil salinization areas [23]. The use of microorganisms was recently investigated for its effectiveness in saline soil remediation, such as in improving soil properties [24,25], decreasing salinity, and alleviating salinity stress [26]. However, many questions remain regarding the mediated processes in saline soil when using a fertilizer with microorganisms, particularly the soil hydraulic properties in both secondary salinization soil and coastal saline soil. Does the application of microbial fertilizer (MF) play a positive role in soil hydraulic properties? Is there a difference between the two types of saline soil? Will an interaction exist between the microbial fertilizer and salinity? All these questions require answers, and clarifying the effects of microbial fertilizer on soil hydraulic properties and the interactive effects of microbial fertilizer and salinity is urgent. Most recent studies have revealed the influence of microorganisms on soil moisture [27,28]; that is, soil salinity interferes with the movement of soil water [29], and microorganisms may regulate soil salt accumulation [30]. Be that as it may, only a few studies have produced convincing evidence for the interactive effects of salinity and microbes on soil hydraulic properties. Available relevant experimental data and evidence that can answer these questions are scarce, at least in secondary salinization and coastal saline soil. Thus, we attempted to use microbial fertilizer to ameliorate both secondary salinization and coastal saline soil and, consequently, reveal the interactive effects of microbial fertilizers and salinity on soil hydraulic properties.

To test our assumption, two types and three levels of saline soil were designated in an incubation experiment to elucidate the process of soil hydraulic properties affected by microbial fertilizer and salinity. The objectives of this study were as follows: to (i) reveal the changes in soil hydraulic properties affected by the application of microbial fertilizer, and (ii) clarify the interactive effects of microbial fertilizer and salinity both on secondary salinization soil and coastal saline soil and the principal driver of the effects on soil hydraulic properties. The results are expected to provide new solutions for strategies to increase hydraulic properties or mitigate the adverse effects of salinity on plants in secondary salinization soils or coastal saline soils. This study not only provides a complex process for the interactive effects of microbial fertilizer and salinity on soil hydraulic properties but further confirms the notion that soil can be improved by applying microbial fertilizer and the practical significance of microbial fertilizer application for soil water conservation and salinity migration in secondary salinization soil and coastal saline soil.

## 2. Materials and Methods

### 2.1. Experimental Soil

Two types of saline soils within the depth of 0–30 cm were sampled in July 2016: a secondary salinization soil from the Vegetables and Flowers Institute of Hohai University test base (31°43′18″ N,118°46′18″ E), Nanjing of Jiangsu Province, Eastern China, and a coastal saline soil from Dongtai City of Jiangsu Province, Eastern China (32°38′29″ N, 120°54′09″ E). The salt in secondary salinization soil was predominantly Ca(NO_3_)_2_ and KNO_3_, while that in coastal saline soil was predominantly NaCl, based on our and others’ previous studies [31,32,33,34,35]. Their electrical conductivity (EC) was about 2.81 dS m^−1^ and 3.25 dS m^−1^. The non-saline soil was collected from farmland at the same soil profile depth adjacent to the saline soil, with an average EC < 2.0 dS m^−1^. The soil properties are listed in Table 1.

### 2.2. Microbial Fertilizer Preparation

The microbial fertilizer used in this study was produced by formulating effective microorganism (EM) liquid, organic fertilizer, rice straw, animal excrement, and molasses. In particular, this microbial fertilizer was a fermentative mixture of effective microorganisms, including more than 80 species of microbes. Moreover, the primary raw materials of the microbial inoculum were organic fertilizer, rice straw, animal excrement, and molasses. Then, the materials were fermented and prepared for more than 30 days. The microbial fertilizer contained organic matter; its total nitrogen was 30% and 5%, and its total (P + K) was 1%. The organic fertilizer was provided by Nanjing Ningliang Bio-fertilizer Co., Ltd., Nanjing, China. The effective microorganisms were provided by Aimule Environmental Biotechnology (Nanjing) Co., Ltd., Nanjing, China. The EMs had a pH of 3.8 and emitted the smell of kvass or fermenting fruit juice (*photosynthetic bacteria*, *yeasts*, *actinomycetes*, and *fermenting fungi*) [36]. the number of contained effective viable bacteria was 10^10^–10^12^ cfu mL^−1^.

### 2.3. Experimental Design

To better compare the differences between soil hydraulic properties and the types and levels of saline soil, we designated the types of saline soils as secondary salinization soil and coastal saline soil. Moreover, the levels of soil salinity in secondary salinization soil were non-saline soil (SS0) and low-salinity soil (SS1), while the levels of soil salinity in coastal saline soil were non-saline soil (CS0) and low-salinity soil (CS1). The SS0, SS1, CS0, and CS1 soils were used as is. By contrast, high-salinity soil (SS2 and CS2, ≈4.0 dS m^−1^) was prepared by adding Ca(NO_3_)_2_ and KNO_3_ to SS1 and adding NaCl to CS1. Each level contained a treatment group that received microbial fertilizer (32.89 g kg^−1^) and a control (0 g kg^−1^, CK), with three replicates for each treatment (Table 2). Microbial fertilizer was mixed into the soil before being placed in pots, and all the soil samples with microbial fertilizer or the control (CK) were used to conduct a 110-day incubation experiment. The experimental pots had an inner diameter of 20 cm and a height of 18 cm.

### 2.4. Soil Physical and Chemical Properties

For soil pH and electrical conductivity (EC) of 1:5 soil: water extracts were obtained using a pH electrode and a conductivity electrode (Mettler-Toledo Ltd., Shanghai, China), respectively. Bulk density was determined using the samples collected with larger ring cutters, with the moist samples being oven-dried at 105 °C to constant weight. Soil available nitrogen content was measured using the alkaline hydrolysis diffusion method [37]. Soil available P was extracted with 0.5 mol L^−1^ NaHCO_3_ [38]. In addition, soil available K was measured using flame photometry, which was extracted with 1 M ammonium acetate [39].

### 2.5. Soil Hydraulic Properties

#### 2.5.1. Soil Water Characteristic Curve

**Figure 1:**
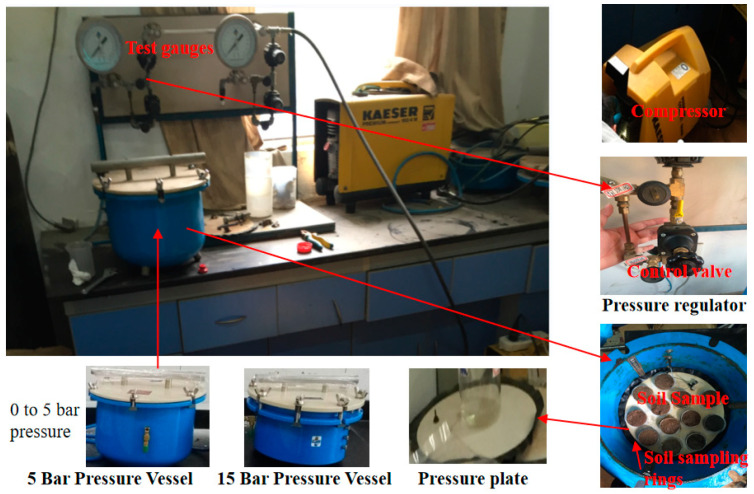
Pressure plate testing using the pressure vessel (5 bar and 15 bar).

Soil water characteristic curves (SWCCs) were determined by using pressure plates (Pressure Vessel 1500, Soil Moisture Equipment Corp., Goleta, CA, USA), following Yang et al. [40]. Suction was successively applied to establish 10 matric potentials of 0, 0.05, 0.1, 0.2, 0.3, 0.5, 1, 5, 10, and 15 bar (100 kPa) (Figure 1). Finally, all the soil samples were maintained at 105 °C until a constant mass was reached. The average value was used to calculate the soil volumetric moisture content at each pressure level [40]. These soil moisture contents and corresponding pressures were used to create each SWCC.

By plotting *θ* against soil matrix suction ($\psi_{m}$), an SWCC was fitted using the Gardner model in accordance with the following formula [40,41]:

(1) $$\theta =A\cdot {\psi_{m}}^{-B},$$

where *θ* is the soil water content (cm^3^ cm^−3^), $\psi_{m}$ is the soil matrix suction (100 kPa), and *A* and *B* are the parameters of the SWCC.

#### 2.5.2. Soil Water Supply Capacity

Soil-specific water capacity is the amount of water released (or absorbed) when the soil water suction increases (or decreases) by one unit. Specific water capacity is frequently used to describe the soil water supply capacity, and it is given as [42]:

(2) $$C\left(\theta \right)=\frac{d\theta }{d\psi }=A\cdot B{\psi_{m}}^{-\left(B+1\right)}$$

where *C*(*θ*) is the soil-specific water capacity (cm^3^ cm^−3^ 100 kPa^−1^).

#### 2.5.3. Soil Water Characteristic Parameters

Soil water characteristic parameters were calculated using the SWCCs, which included moisture at saturation (*θ_sat_*, defined as the volumetric soil water content when the matric potential is −0 bar), field capacity (*θ_fc_*, defined as the volumetric soil water content when the matric potential is −0.3 bar), capillary fracture (*θ_cp_*, about 65% of *θ_fc_*), the wilting coefficient (*θ_wc_*, defined as the volumetric soil water content when the matric potential is −15 bar), and the hygroscopic coefficient (*θ_hyg_*, *θ_hyg_* is *θ_wc_* divided by 1.5 to 2.0 times) [40].

#### 2.5.4. Soil Water Availability

Soil water availability included gravity water (GM, the value of *θ_sat_* minus *θ_fc_*), available water (AW, the value of *θ_fc_* minus *θ_wc_*), readily available water (RAW, the value of *θ_fc_* minus *θ_cp_*), and unavailable water (UAW, the water less than *θ_wc_*) [40,43,44].

The formulas for the coefficient of determination (R^2^), mean error (ME, cm^3^ cm^−3^), and root mean square error (RMSE, cm^3^ cm^−3^) are as follows [45,46]:

(3) $$RMSE=\sqrt{\frac{\sum_{k=1}^{n}{\left(P_{k}-M_{k}\right)}^{2}}{n}},$$

(4) $$ME=\frac{1}{n}\sum_{k=1}^{n}\left(P_{k}-M_{k}\right),$$

(5) $$R^{2}=1-\frac{\sum_{k=1}^{n}{\left(P_{k}-M_{k}\right)}^{2}}{\sum_{k=1}^{n}{\left(M_{k}-\bar{M}\right)}^{2}},$$

where *M_k_* and *P_k_* are the measured and predicted values, and *k* = 1, 2, 3, …, *n*; $\bar{M}$ is the mean of *M_k_*; and *n* is the number of observations.

### 2.6. Statistical Analysis

Statistical analysis for a randomized plot design was performed using SPSS 17.0. When statistical significance (*p* < 0.05) was detected, Duncan’s multiple range tests were performed on the mean values. The figures were drawn using Origin 2021 (OriginLab, Northampton, MA, USA). The relationships between soil hydraulic properties and microbial fertilizer and salinity were analyzed using redundancy analysis (RDA). The microbial fertilizer and salinity data used in the regression analysis were normalized values, which were log (*x* + 1) transformed before the analysis to meet the normality and homogeneity of variance [47].

## 3. Results

### 3.1. Changes in Soil Water Characteristic Curves

**Figure 2:**
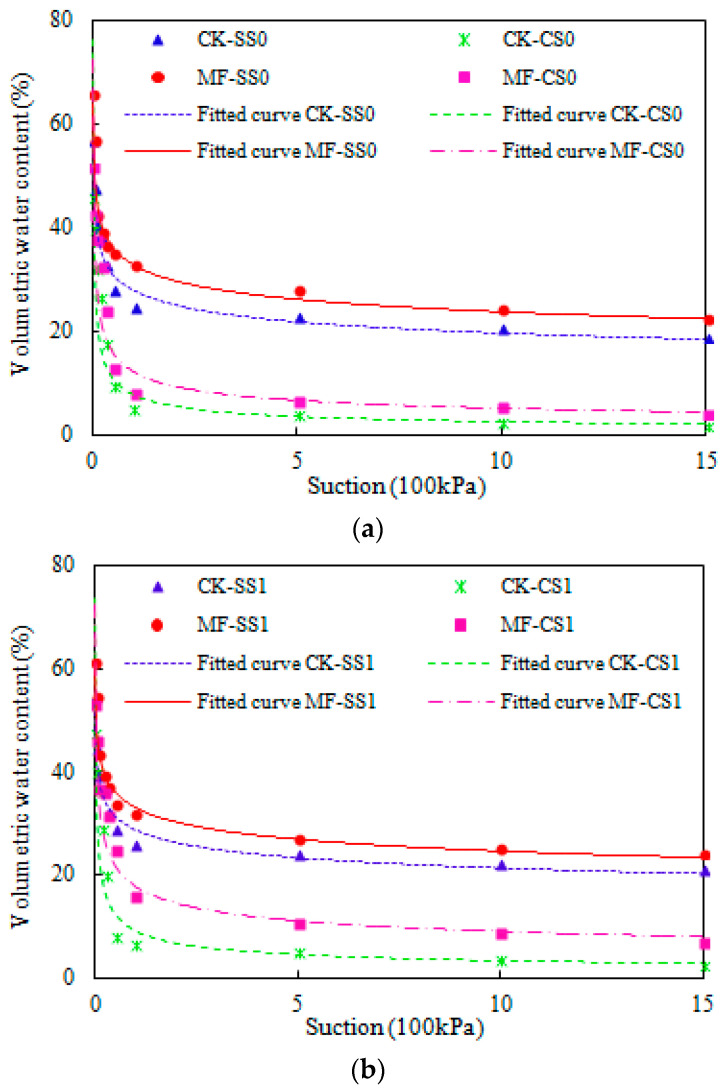

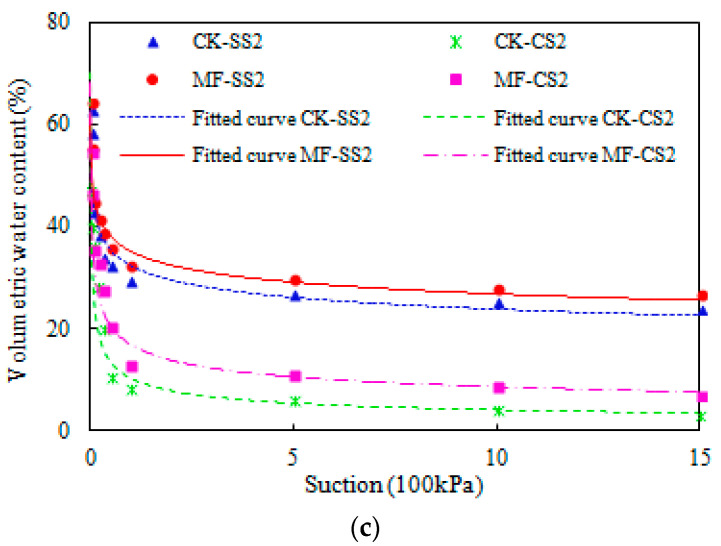
Soil water characteristic curves of different saline soil types within the suction range of 0–15 (100 kPa). (**a**) SS0 (secondary salinization soil with non-saline) and CS0 (coastal saline soil with non-saline); (**b**) SS1 (secondary salinization soil with low salinity) and CS1 (coastal saline soil with low-salinity); (**c**) SS2 (secondary salinization soil with high salinity) and CS2 (coastal saline soil with high-salinity).

The SWCCs of the different types of saline soil are shown in Figure 2a–c, and the parameters of each treatment fitted by the Gardner model are provided in Table 3. As depicted in Figure 2a–c, the water content of all the treatments presented a decreasing trend with an increase in soil water suction, which decreases sharply before 0.5 bar (100 kPa) and, subsequently, changes more slowly at 1–15 bar (100 kPa). The application of microbial fertilizer significantly affects both secondary salinization soil and coastal saline soil. In non-saline soil, the SWCCs of the microbial fertilizer application in MF-SS0 and MF-CS0 were higher than those in CK-SS0 and CK-CS0 (Figure 2). The slope of the SWCCs demonstrated an increased variation with the application of microbial fertilizer, and this increase in variability resulted in corresponding changes in soil water holding capacity. Moreover, the Gardner model parameters (A) and (A·B) in MF-SS0 and MF-CS0 were significantly increased by 15.13% and 36.86% (A), and 8.39% and 18.83 (A·B), respectively, compared with CK-SS0 and CK-CS0 (Table 3), indicating that soil water holding capacity with microbial fertilizer applied to each type of saline soil was higher than that in CK, and soil water holding capacity in secondary salinization soil was higher than that in coastal saline soil. From the SWCCs and the parameters (A and A·B), the order of soil water holding capacity can be concluded as follows: MF-SS0 > CK-SS0 > MF-CS0 > CK-CS0 (Figure 2a). Similar changes occurred in low-salinity soil and high-salinity soil, the order of soil water holding capacity is MF-SS1 > CK-SS1 > MF-CS1 > CK-CS1 and MF-SS2 > CK-SS2 > MF-CS2 > CK-CS2, respectively (Figure 2b,c).

**Figure 3:**
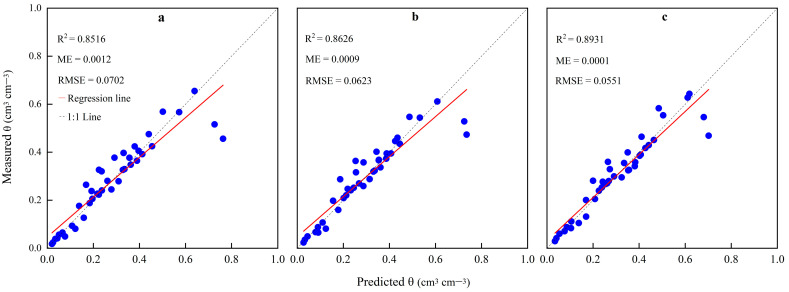
Comparison of the measured and predicted soil water contents (*θ*) for (**a**) non-saline soil, (**b**) low-salinity soil, and (**c**) high-salinity soil.

The SWCCs were fitted using the Gardner model, and the correlation coefficients of the SWCCs were R^2^ > 0.91 (Table 3), indicating that the data that fitted the soil water characteristic curves were closely correlated to the measured data. Moreover, the *θ* values measured and predicted by the Gardner model for each saline level are shown in Figure 3. A general agreement in the non-saline, low-salinity, and high-salinity soils was observed between the predicted and measured *θ* values because the R^2^ value of the linear regression was within the range of 0.8516–0.8931, the ME was within the range of 0.0001–0.0012 cm^3^ cm^−3^, and the RMSE was within the range of 0.0551–0.0702 cm^3^ cm^−3^, where the latter two were close to zero. The R^2^ value of high-salinity soil was higher than that of non-saline soil (Figure 3), indicating that the saline levels in the soil affected the SWCCs, and the high-salinity soil was better.

### 3.2. Change in Soil Water Characteristic Parameters

Soil water characteristic parameters were significantly affected by microbial fertilizer and salinity (Table 4). Microbial fertilizer exerted significant effects on soil water characteristic parameters (e.g., *θ_sat_*, *θ_fc_*, *θ_cp_*, *θ_wc_*, and *θ_hyg_*) both in secondary salinization soil and coastal saline soil. All the soil water characteristic parameters with the application of microbial fertilizer were higher than those of CK. In particular, applied microbial fertilizer increased the *θ_sat_*, *θ_fc_*, *θ_cp_*, *θ_wc_*, and *θ_hyg_* parameters in secondary salinization soil by 0.02–18.91% (Table 4). By contrast, applied microbial fertilizer in coastal saline soil was significantly higher than that in secondary salinization soil, increasing by 11.62–181.88% (Table 4). The types and levels of saline soil also significantly affected the soil water characteristic parameters (Table 4). The elevated levels of saline soil significantly increased the soil water characteristic parameters in coastal saline soil and high-salinity secondary salinization soil. By contrast, only parameter *θ_hyg_* increased in low-salinity secondary salinization soil. Basically, *θ_fc_* and *θ_wc_* can reflect soil water holding capacity. Our data demonstrated that *θ_fc_* and *θ_wc_* in coastal saline soil increased by 12.20% and 31.32% (CS1), and by 13.90% and 62.64% (CS2) compared with CS0. Moreover, *θ_fc_* and *θ_wc_* in high-salinity secondary salinization soil were increased by 3.64% and 27.53% (SS2), respectively. Meanwhile, only *θ_wc_* increased in low-salinity secondary salinization soil by 11.29% (SS1) compared with SS0.

### 3.3. Changes in Soil Water Supply Capacity

Soil water supply capacity refers to the ability of soil to supply water to plants under certain conditions [42]. The *C*(*θ*) value for each soil water suction is calculated in Table 5. The specific water capacity decreased with increasing soil water suction. This finding is attributed to the fact that changes in suction caused larger pore drainage in the low-suction section, and the corresponding specific water capacity is larger. By contrast, soil water pore aperture size becomes smaller with increased suction, and the corresponding specific water capacity is smaller. When the suction was 0.5 bar (100 kPa), the soil lost water rapidly in all the treatments, and the *C*(*θ*) values were 10^2^ (most of the secondary salinization soil) or 10^1^ (coastal saline soils) orders of magnitude (Table 5).

The application of microbial fertilizer in specific water capacity was higher than in no application, indicating that microbial fertilizer could increase soil water supply capacity, increasing the drought resistance of the soil. In particular, the specific water capacity was significantly increased in MF-SS0 and MF-SS1 by 0.02–7.40% and 0.02–14.53%, respectively, compared with that in CK, while no significant difference was noted in MF-SS2 (Table 5). Moreover, specific water capacity decreased in CS0, CS1, and CS2 when the suction was lower (<0.2 bar). In comparison, the application of microbial fertilizer gradually played a role with an increase in suction (0.3–15 bar). The soil-specific water capacity in MF-CS0, MF-CS1, and MF-CS2 was increased by 0.04–1.83%, 0.08–2.34%, and 0.06–1.12%, respectively, compared with that in CK (Table 5). Salinity also exerted significant and identical effects on soil-specific water capacity. Soil-specific water capacity was slightly higher than that in CK with an increase in salinity when suction was higher (>0.3 bar) and only found in CK-SS2, CK-CS2, and CK-CS1. However, soil-specific water capacity was lower than that in CK with an increase in salinity in CK-SS1 and CK-SS2 (<0.3 bar), CK-CS1 (<0.1 bar), and CK-CS2 (<0.2 bar) when suction was lower. Moreover, soil-specific water capacity was lower than that in CK with an increase in salinity in MF-SS1, MF-SS2, and MF-CS2. Meanwhile, it was lower than that in CK in MF-CS1 with lower suction (<0.1 bar) because the application of microbial fertilizer plays a leading role and exhibits better water supply capacity and drought resistance.

### 3.4. Changes in Soil Water Availability

The application of microbial fertilizer increased available water, readily available water (except SS0), and unavailable water in secondary salinization soils and coastal saline soils. Meanwhile, no significant relationship was found for gravity water (Table 6). Notably, available soil water for the application of microbial fertilizer in MF-SS1 and MF-SS2 was increased by 14.12% and 19.80%, respectively, compared with that in CK (Table 6). Moreover, the soil available water in MF-CS0, MF-CS1, and MF-CS2 increased by 24.81%, 43.15%, and 20.46%, respectively, compared with that of CK. For soil readily available water after microbial fertilizer application, the value was increased by 10.67%, 15.49%, and 14.14% in SS and increased by 35.17%, 59.83%, and 38.03% in CS compared with that in CK (Table 6). These findings indicate that applying microbial fertilizer can increase the soil’s available and readily available water, and the effects on coastal saline soils are greater than those on secondary salinization soils. As salinity increased, soil available water increased by 10.0% and 8.29% in coastal saline soils, but it decreased by 19.55% and 28.02% in secondary salinization soils. Similarly, soil readily available water increased in coastal saline soils (by 12.16% and 13.78%) and secondary salinization soils (SS2, 3.64%) but decreased in secondary salinization soils (SS1, 1.99%) (Table 6).

### 3.5. Relationships among Microbial Fertilizer, Salinity, and Soil Hydraulic Properties

**Figure 4:**
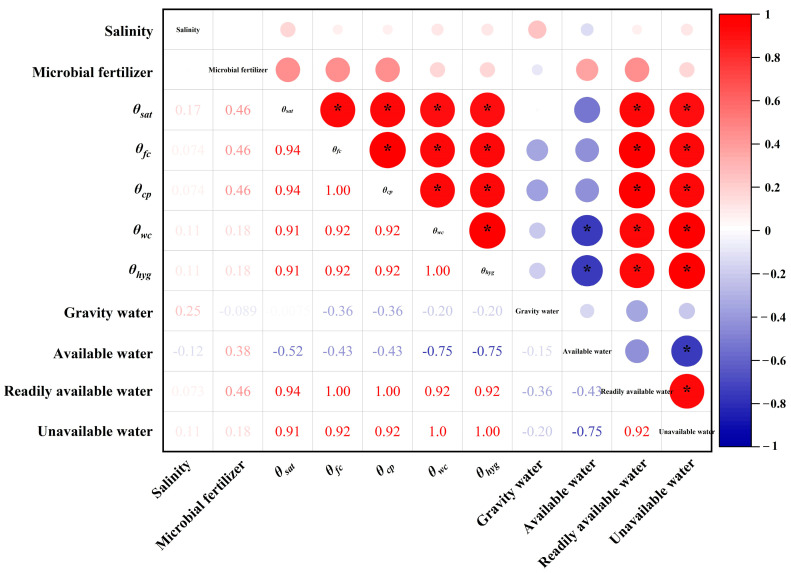
Correlation heat map with significant marks between microbial fertilizer and salinity and soil hydraulic properties. Note: red represents positive correlations, while blue represents negative correlations. * *p* < 0.05.

The correlation analysis among microbial fertilizer, salinity, and soil hydraulic properties is depicted in Figure 4. Microbial fertilizer exhibited a positive correlation with *θ_sat_*, *θ_fc_*, *θ_cp_*, *θ_wc_*, *θ_hyg_*, available water, readily available water, and unavailable water but a negative correlation with gravity water (Figure 4), indicating that the application of microbial fertilizer increased *θ_sat_*, *θ_fc_*, *θ_cp_*, *θ_wc_*, and *θ_hyg_* and soil water availability. That is, the application of microbial fertilizer can condition plants to resist wilting under water deficits and salinity stress. In particular, it improves *θ_fc_*, available water, and readily available water; this condition is conducive to the absorption of available water by plants. Notably, salinity exhibited a negative correlation with soil available water (Figure 4), indicating that increasing or excessive salinity exerted an inhibitory effect on soil available water by altering soil water transport. In addition, available water presented a significant and negative correlation with *θ_wc_* (*p* < 0.05). That is, an increase in the wilting coefficient resulted in less soil available water. However, what seems clear is that a significant amount of water stored by this soil may not be available to plant roots.

### 3.6. Interpretations of Soil Hydraulic Properties and Microbial Fertilizer and Salinity Using RDA

**Figure 5:**
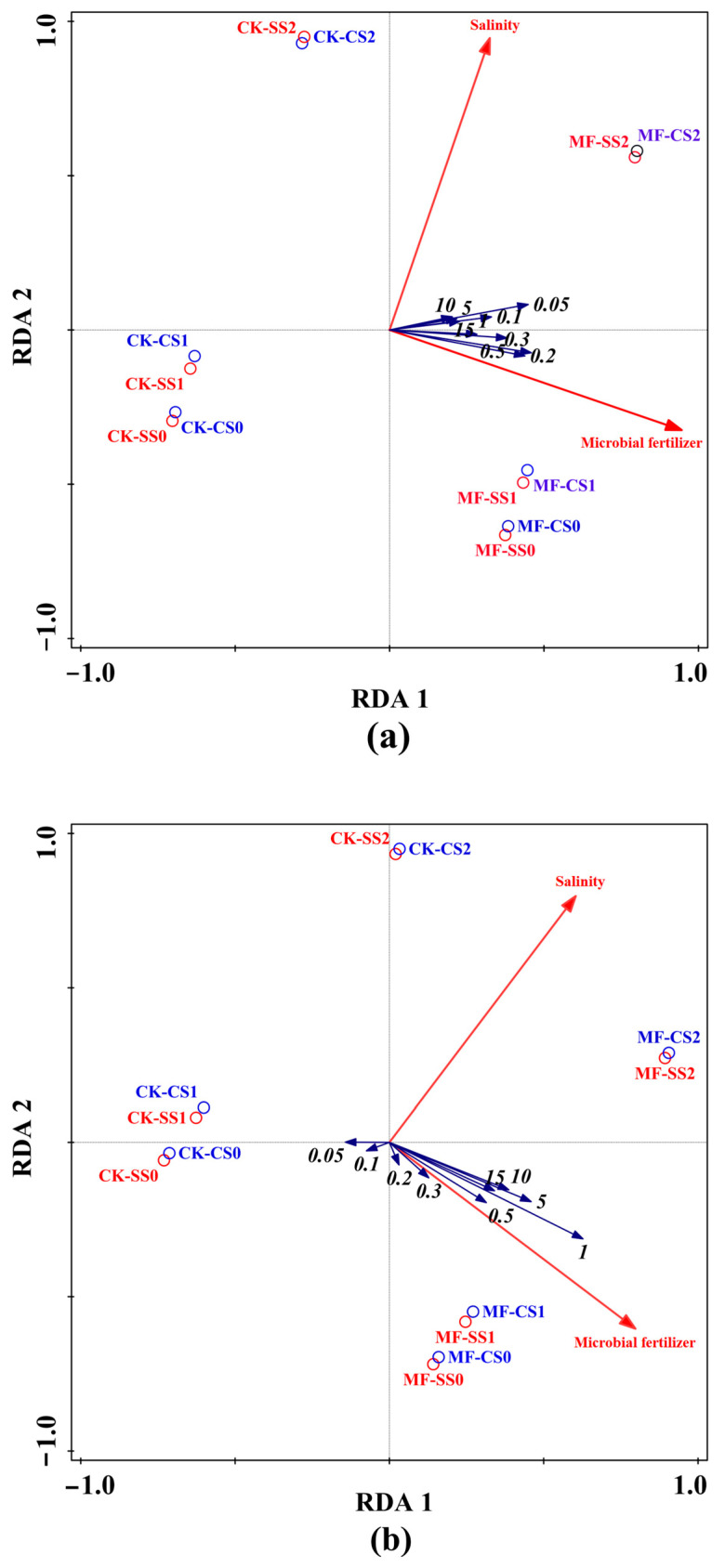

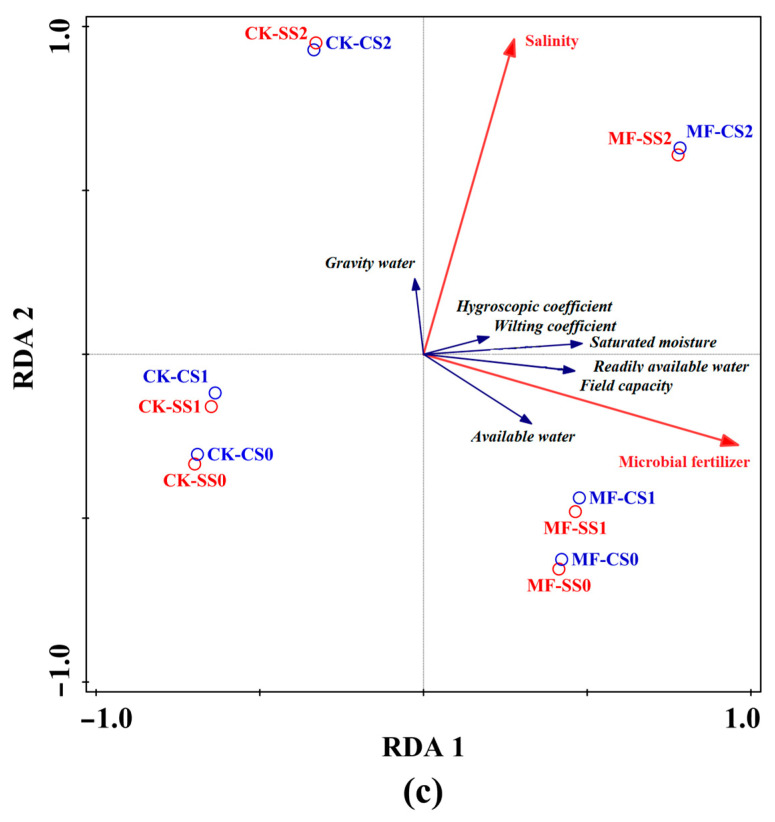
RDA illustrating the relationships between soil hydraulic properties and microbial fertilizer and salinity. Note: the numbers 0.05, 0.1, 0.2, 0.3, 0.5, 1, 5, 10, and 15 represent the soil water holding capacity and specific water capacity of each suctions (100 kpa).

The redundancy analysis (RDA) indicated that the microbial fertilizer and salinity altered soil water holding capacity (Figure 5a). In the ordination diagrams, soil water holding capacity under each soil water suction and microbial fertilizer exhibited the strongest positive correlations, while salinity presented positive correlations with soil water holding capacity (Figure 5a).

The RDA showed that the application of microbial fertilizer exhibited a significant positive correlation with soil water supply capacity after lower soil suction (>0.1 bar). By comparison, salinity only presented a positive correlation with soil water supply capacity at higher suction (>0.5 bar) (Figure 5b). However, no similar relationship was observed in lower suction and low-salinity soil (CK-SS1).

Regardless of soil type, the RDA indicated that soil water characteristic parameters increased with increasing salinity (except SS1) and the application of microbial fertilizer. Moreover, the soil water characteristic parameters presented significant positive correlations with microbial fertilizer while only demonstrating positive correlations with salinity (Figure 5c). In our study, the RDA indicated that soil available water, readily available water, and unavailable water were positively correlated with microbial fertilizer; moreover, soil gravity water, readily available water, and unavailable water were positively correlated with salinity (Figure 5c). However, soil available water was negatively correlated with salinity.

## 4. Discussion

### 4.1. Interactive Effects of Microbial Fertilizer and Salinity on Soil Water Characteristic Curves

Our results showed that applying microbial fertilizer improved soil water holding capacity compared with that of CK. However, this effect depended on the types and levels of saline soils. In non-saline soil, microbial fertilizer exerts a greater effect on secondary salinization soil than coastal saline soil. In low-salinity and high-salinity soil, the effect on coastal saline soil is greater than that of secondary salinization soil. Thus, the microbial fertilizer and salinity interaction affects soil water holding capacity. These results are consistent with those obtained in a previous study, which reported that soil water holding capacity was significantly increased compared with that of the control when fertilizer was applied [48]. Similar findings were observed by Duan et al. [49], who indicated that bio-organic fertilizers can improve soil physical structure and hydraulic characteristics, resulting in improved soil water holding capacity. Moreover, our data demonstrated that soil water holding capacity increased with increasing salinity levels. This result was emphasized by Zhang et al. [50], who reported an interaction effect between changes in soil ion content and the movement of soil pore water in saline soil, where soil water holding capacity increased with increasing soil ion content. Similarly, Sun et al. [51] found that more Na^+^, K^+^, Cl^−^, and other salt ions in vivo were absorbed and accumulated by plants in saline soil. This condition can actively improve water absorption capacity and water retention capacity.

### 4.2. Interactive Effects of Microbial Fertilizer and Salinity on Soil Water Characteristic Parameters

Soil *θ_sat_*, *θ_fc_*, and *θ_cp_* were negatively correlated with an increase in salinity in SS1, while a significant positive relationship was found in the other types of saline soil (Table 4). The relationship between *θ_sat_*, *θ_fc_*, *θ_cp_*, *θ_wc_*, and *θ_hyg_* and microbial fertilizer was more significant in coastal saline soil than in secondary salinization soil. The relationship between *θ_sat_*, *θ_fc_*, *θ_cp_*, *θ_wc_*, and *θ_hyg_* and salinity was significant in SS2 and CS2, which contained lower salt levels than SS1 and CS1. Similar findings were also confirmed by Chen et al. [52], who indicated that the functional bacteria in bio-organic fertilizer can promote the formation of soil aggregates, which can increase water content at both *θ_fc_* and *θ_wc_*. The current research indicated that *θ_sat_*, *θ_fc_*, and *θ_wc_* were highly significantly responsive to the combined application fertilizer [53]. Some studies have indicated that soil water content at *θ_wc_* increased with increasing soil salinity [54]. The results were in agreement with the findings of Sun et al. [51], who reported that soil with moderate salinity can enhance *θ_fc_* water retention.

### 4.3. Interactive Effects of Microbial Fertilizer and Salinity on Soil Water Supply Capacity

The interaction between microbial fertilizer and salinity significantly influenced the specific water capacity of all soil samples. Our results proved that the application of microbial fertilizer has significant positive correlations with soil water supply capacity after lower soil suction (>0.1 bar). By comparison, salinity only exhibits positive correlations with soil water supply capacity at higher suction (>0.5 bar). These results are in line with a previous report by Kuila and Ghosh [55], which showed that application of *arbuscular mycorrhizal fungi* can increase plant water uptake and soil water holding capacity and increase tolerance to soil salinity stresses. Noticeably, our results indicated that the soil water supply capacity increased with increasing salinity at higher suction. Interestingly, Zhang et al. [50] concluded that soil residual water content increased with increasing soil ion content, improving soil water holding capacity while reducing soil water supply capacity. This finding is attributed to the lower thickness of the water attached to the soil surface (adsorbed water) under higher soil water salinity levels, resulting in a lower conducting soil water content [56].

### 4.4. Interactive Effects of Microbial Fertilizer and Salinity on Soil Water Availability

Soil water availability is also influenced by the interaction between microbial fertilizer and salinity. Our study indicated that soil available water, readily available water, and unavailable water were positively correlated with microbial fertilizer, and soil gravity water, readily available water, and unavailable water were positively correlated with salinity. However, soil available water exhibits a negative correlation with salinity levels. This finding is attributed to the fact that the effects of specific salt ion toxicity dominated, and with an increase in salinity, water extraction by plants was significantly less than that of the predicted values based on the total soil water potential [57]. However, in any case, microbial fertilizer can increase soil water availability in secondary salinization soils and coastal saline soils, which are considered readily available water for plants. These findings were also confirmed by Li et al. [58], who found that soil available water significantly responds to fertilizer application.

Moreover, a previous study revealed that soil water holding capacity, *θ_fc_*, readily available water, and water absorption were increased with an increase in soil salinity [51]. From a practical point of view, however, salinity adversely affects plants. Excess salts affect the uptake of nutrients and water by plants, and changes in the soil environment can affect microbial activities [59], interfering with the normal and microbe-mediated soil processes [60]. Consequently, plant growth is affected, although parts of the soil water characteristic parameters increase with salt content.

The present study highlights the importance of a direct link between microbial fertilizer and soil hydraulic properties. However, different salt levels significantly affect soil hydraulic properties, and thus, we explained and discussed why salinity affects soil hydraulic properties and the interaction between salinity and microbial fertilizer. Despite all these inconsistent findings and controversy, in any case, our results from a carefully designed and measured experiment suggested that the application of microbial fertilizer was the principal driver for improving the hydraulic properties of saline soil. As a whole, our results indicated that microbial fertilizer contributes directly to soil water holding capacity, soil water characteristic parameters, soil water supply capacity, and soil water availability. This outcome encourages further studies and methodological development to improve our understanding of the relationship between microbial fertilizer and its direct effect on soil hydraulic properties.

## 5. Conclusions

To our knowledge, this study is a rare report on the interactive effect of microbial fertilizer and salinity on both secondary salinization and coastal saline soil and its effects on the interactions among soil hydraulic properties. Soil water holding capacity improved with the application of microbial fertilizer and increasing salinity levels. The soil water characteristic parameters increased with microbial fertilizer, but they increased with salinity only in coastal saline and high-salinity secondary salinization soil. In addition, the soil-specific water capacity of microbial fertilizer increased in secondary salinization soils (non- and low-) and coastal saline soils by 0.02–14.53% and 0.04–2.34% compared with CK while only demonstrating a positive correlation with salinity at higher suction (>0.5 bar). Moreover, available, readily available, and unavailable water were positively correlated with microbial fertilizer (increased above 10.67%), and gravity, readily available, and unavailable water were positively correlated with salinity. In contrast, available water in secondary salinization soils was negatively correlated with salinity. However, although the increase in salt content can enhance parts of the soil’s hydraulic properties, it exerts an adverse effect on plant growth and soil properties. In any case, microbial fertilizer was the principal driver for improving the hydraulic properties of both secondary salinization and coastal saline soils. A conclusion could be drawn from the above results that microbial fertilizer should be suggested to increase hydraulic properties or mitigate the adverse effects of salinity on plants in secondary salinization soils or coastal saline soils.

## Figures and Tables

**Table 1:** Properties of secondary salinization soil and coastal saline soil.

Soil Parameters	Non-Saline Secondary Soil	Secondary Salinization Soil	Non-Saline Coastal Soil	Coastal Saline Soil	Significance
Bulk density (g cm^−3^)	1.33	1.38	1.22	1.24	n.s.
EC (dS m^−1^)	1.19	2.81	1.44	3.25	n.s.
pH	5.62	4.98	8.51	8.44	a
Available N (mg kg^−1^)	205.98	152.15	25.09	41.02	a
Available P (mg kg^−1^)	257.64	291.50	35.49	70.40	a
Available K (mg kg^−1^)	310.26	270.91	228.36	262.08	a

n.s. and a represent no significance and a significant difference at *p* < 0.01, respectively. EC: electrical conductivity.

**Table 2:** Experimental design.

Microbial Fertilizer (g kg^−1^)	Secondary Salinization Soil Level	Coastal Saline Soil Level
0	SS0	CS0
	SS1	CS1
	SS2	CS2
32.89	SS0	CS0
	SS1	CS1
	SS2	CS2

**Table 3:** Gardner fitting parameters and equations for soil water characteristic curves.

Microbial Fertilizer	Saline Soil	A	B	R^2^	Fitting Equation
CK	SS0	0.2787	0.1529	0.9660	*θ* = 0.2787·*ψ_m_*^−^^0.1529^
	SS1	0.2873	0.1308	0.9680	*θ* = 0.2873·*ψ_m_*^−^^0.1308^
	SS2	0.3245	0.1342	0.9254	*θ* = 0.3245·*ψ_m_*^−^^0.1342^
	CS0	0.0771	0.4866	0.9313	*θ* = 0.0771·*ψ_m_*^−^^0.4866^
	CS1	0.0915	0.4424	0.9164	*θ* = 0.0915·*ψ_m_*^−^^0.4424^
	CS2	0.1043	0.4047	0.9359	*θ* = 0.1043·*ψ_m_*^−^^0.4047^
Microbial fertilizer	SS0	0.3284	0.1415	0.9673	*θ* = 0.3284·*ψ_m_*^−^^0.1415^
	SS1	0.3306	0.1293	0.9716	*θ* = 0.3306·*ψ_m_*^−^^0.1293^
	SS2	0.3529	0.1191	0.9603	*θ* = 0.3529·*ψ_m_*^−^^0.1191^
	CS0	0.1221	0.3785	0.9235	*θ* = 0.1221·*ψ_m_*^−^^0.3785^
	CS1	0.1782	0.2979	0.9474	*θ* = 0.1782·*ψ_m_*^−^^0.2979^
	CS2	0.1700	0.2945	0.9584	*θ* = 0.1700·*ψ_m_*^−^^0.2945^

**Table 4:** Soil water characteristic parameters.

Microbial Fertilizer	Saline Soil	*θ_sat_* (cm^3^ cm^–3^)	*θ_fc_* (cm^3^ cm^–3^)	*θ_cp_* (cm^3^ cm^–3^)	*θ_wc_* (cm^3^ cm^–3^)	*θ_hyg_* (cm^3^ cm^–3^)
CK	SS0	0.5670	0.3295	0.2142	0.1878	0.1174
	SS1	0.5440	0.3230	0.2100	0.2090	0.1306
	SS2	0.6278	0.3415	0.2220	0.2395	0.1497
	CS0	0.4563	0.1762	0.1145	0.0182	0.0114
	CS1	0.4733	0.1977	0.1285	0.0239	0.0149
	CS2	0.4690	0.2007	0.1305	0.0296	0.0185
Microbial fertilizer	SS0	0.6550	0.3645	0.2369	0.2233	0.1396
	SS1	0.6116	0.3730	0.2425	0.2429	0.1518
	SS2	0.6433	0.3898	0.2534	0.2676	0.1673
	CS0	0.5160	0.2383	0.1549	0.0411	0.0257
	CS1	0.5283	0.3160	0.2054	0.0672	0.0420
	CS2	0.5463	0.2770	0.1801	0.0709	0.0443

**Table 5:** Specific water capacity.

Microbial Fertilizer	Saline Soil	Soil Suction (100 kPa)								
		0.05	0.1	0.2	0.3	0.5	1	5	10	15
CK	SS0	1.3470	0.6058	0.2724	0.1707	0.0947	0.0426	0.0067	0.0030	0.0019
	SS1	1.1127	0.5081	0.2321	0.1467	0.0823	0.0376	0.0061	0.0028	0.0018
	SS2	1.3005	0.5925	0.2699	0.1704	0.0955	0.0435	0.0070	0.0032	0.0020
	CS0	3.2221	1.1498	0.4103	0.2246	0.1051	0.0375	0.0034	0.0012	0.0007
	CS1	3.0483	1.1216	0.4127	0.2300	0.1101	0.0405	0.0040	0.0015	0.0008
	CS2	2.8371	1.0716	0.4047	0.2290	0.1117	0.0422	0.0044	0.0017	0.0009
Microbial fertilizer	SS0	1.4209	0.6441	0.2920	0.1838	0.1026	0.0465	0.0074	0.0034	0.0021
	SS1	1.2580	0.5751	0.2629	0.1663	0.0934	0.0427	0.0069	0.0032	0.0020
	SS2	1.2001	0.5525	0.2544	0.1616	0.0912	0.0420	0.0069	0.0032	0.0020
	CS0	2.8715	1.1044	0.4248	0.2429	0.1201	0.0462	0.0050	0.0019	0.0011
	CS1	2.5924	1.0544	0.4288	0.2534	0.1306	0.0531	0.0066	0.0027	0.0016
	CS2	2.4211	0.9870	0.4024	0.2381	0.1229	0.0501	0.0062	0.0025	0.0015

**Table 6:** Soil water availability.

Microbial Fertilizer	Saline Soil	Gravity Water (cm^3^ cm^–3^)	Available Water (cm^3^ cm^–3^)	Readily Available Water (cm^3^ cm^–3^)	Unavailable Water (cm^3^ cm^–3^)
CK	SS0	0.2375	0.1417	0.1153	0.1878
	SS1	0.2210	0.1140	0.1130	0.2090
	SS2	0.2863	0.1020	0.1195	0.2395
	CS0	0.2801	0.1580	0.0617	0.0182
	CS1	0.2756	0.1738	0.0692	0.0239
	CS2	0.2683	0.1711	0.0702	0.0296
Microbial fertilizer	SS0	0.2905	0.1412	0.1276	0.2233
	SS1	0.2386	0.1301	0.1305	0.2429
	SS2	0.2535	0.1222	0.1364	0.2676
	CS0	0.2777	0.1972	0.0834	0.0411
	CS1	0.2123	0.2488	0.1106	0.0672
	CS2	0.2693	0.2061	0.0969	0.0709

## Data Availability

Data are contained within the article.
